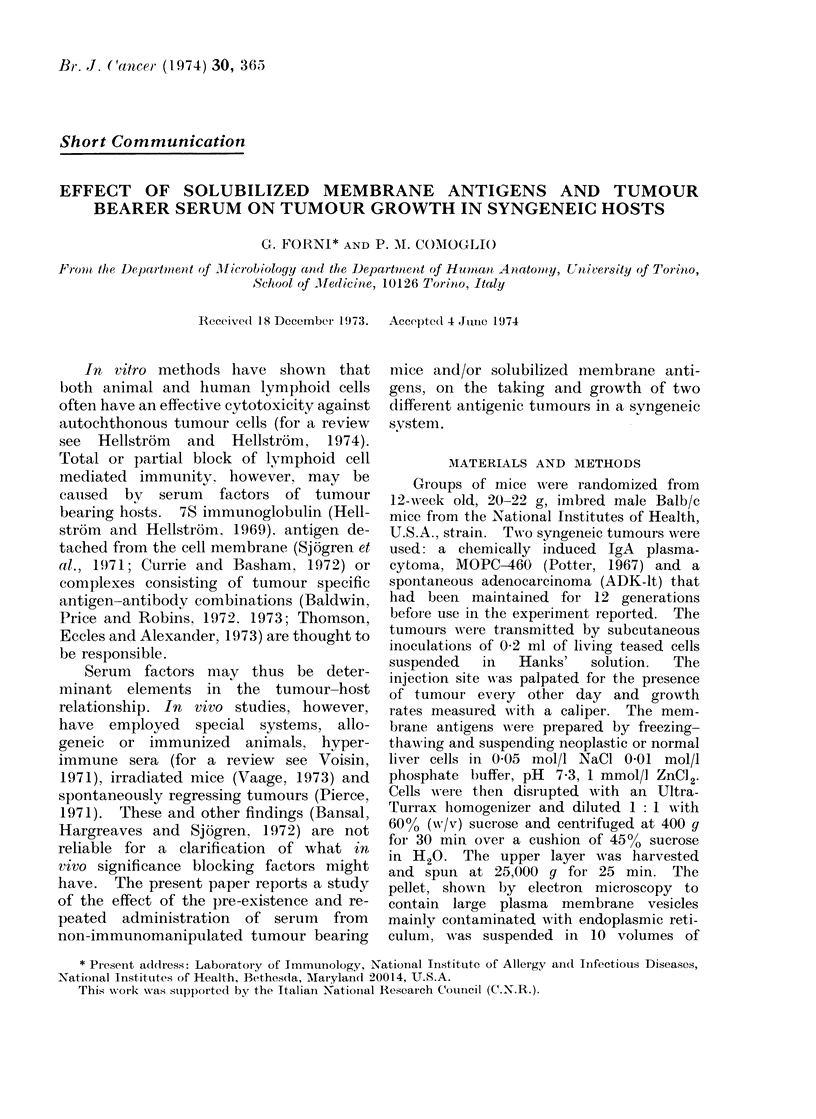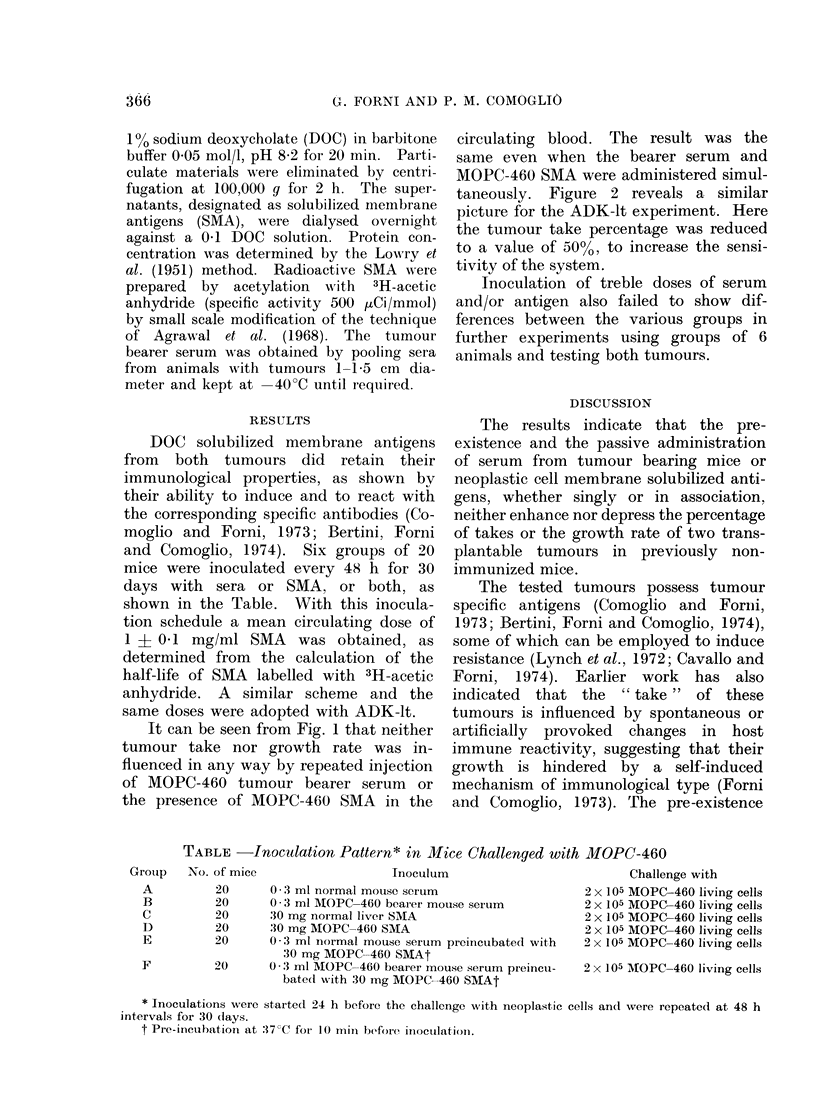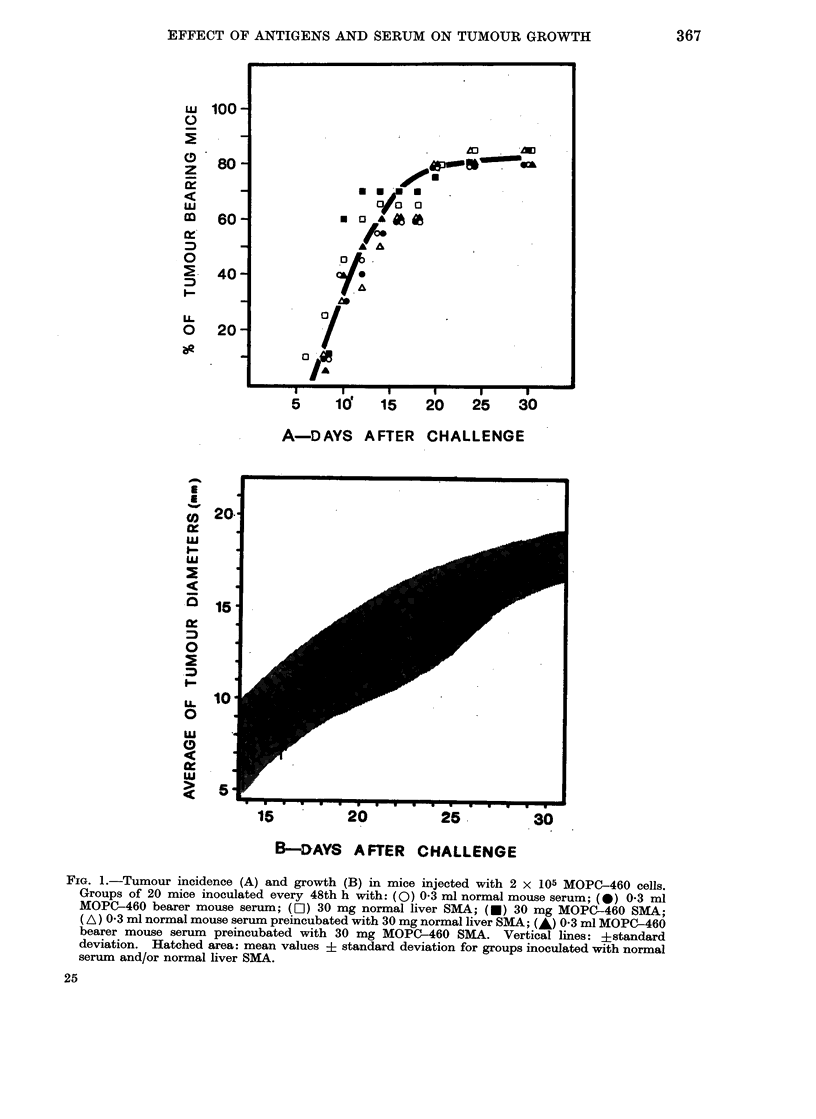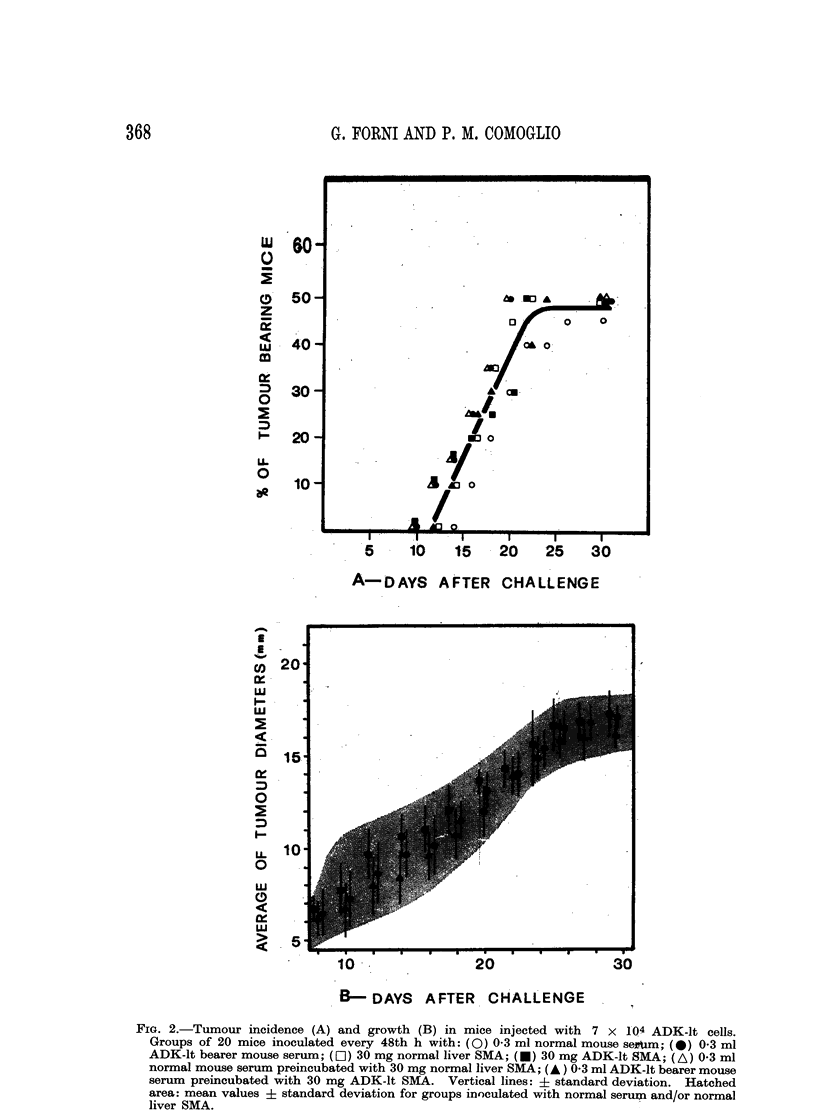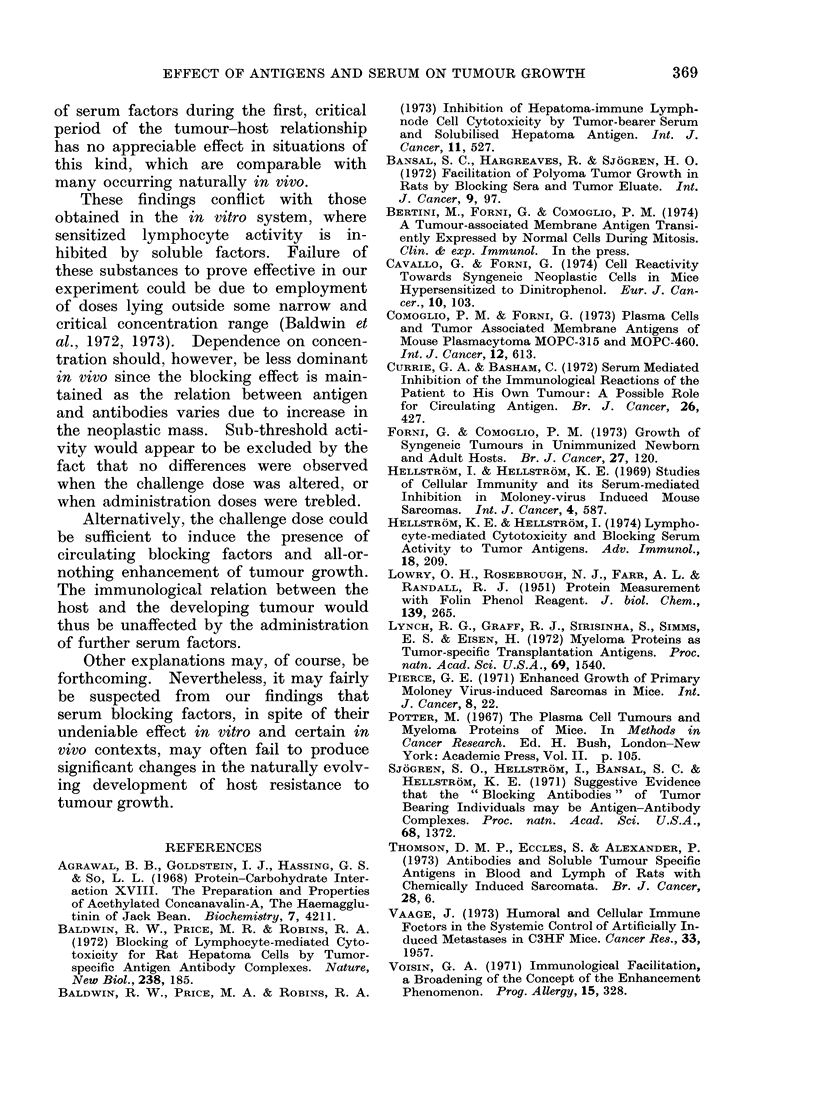# Effect of solubilized membrane antigens and tumour bearer serum on tumour growth in syngeneic hosts.

**DOI:** 10.1038/bjc.1974.206

**Published:** 1974-10

**Authors:** G. Forni, P. M. Comoglio


Br. J. Cancer (1974) 30, 365

### Short Communication

### EFFECT OF SOLUBILIZED MEMBRANE ANTIGENS AND TUMOUR BEARER SERUM ON TUMOUR GROWTH IN SYNGENEIC HOSTS

G. FORNI* AND P. M. COMOGLIO

*From the Department of Microbiology and the Department of Human Anatomy, University of Torino, School of Medicine, 10126 Torino, Italy*

Received 18 December 1973. Accepted 4 June 1974

*In vitro* methods have shown that both animal and human lymphoid cells often have an effective cytotoxicity against autochthonous tumour cells (for a review see Hellström and Hellström, 1974). Total or partial block of lymphoid cell mediated immunity, however, may be caused by serum factors of tumour bearing hosts. 7S immunoglobulin (Hellström and Hellström, 1969), antigen detached from the cell membrane (Sjögren *et al.*, 1971; Currie and Basham, 1972) or complexes consisting of tumour specific antigen–antibody combinations (Baldwin, Price and Robins, 1972, 1973; Thomson, Eccles and Alexander, 1973) are thought to be responsible.

Serum factors may thus be determinant elements in the tumour–host relationship. *In vivo* studies, however, have employed special systems, allogeneic or immunized animals, hyperimmune sera (for a review see Voisin, 1971), irradiated mice (Vaage, 1973) and spontaneously regressing tumours (Pierce, 1971). These and other findings (Bansal, Hargreaves and Sjögren, 1972) are not reliable for a clarification of what *in vivo* significance blocking factors might have. The present paper reports a study of the effect of the pre-existence and repeated administration of serum from non-immunomanipulated tumour bearing mice and/or solubilized membrane antigens, on the taking and growth of two different antigenic tumours in a syngeneic system.

### MATERIALS AND METHODS

Groups of mice were randomized from 12-week old, 20–22 g, imbred male Balb/c mice from the National Institutes of Health, U.S.A., strain. Two syngeneic tumours were used: a chemically induced IgA plasmacytoma, MOPC–460 (Potter, 1967) and a spontaneous adenocarcinoma (ADK-lt) that had been maintained for 12 generations before use in the experiment reported. The tumours were transmitted by subcutaneous inoculations of 0·2 ml of living teased cells suspended in Hanks' solution. The injection site was palpated for the presence of tumour every other day and growth rates measured with a caliper. The membrane antigens were prepared by freezing–thawing and suspending neoplastic or normal liver cells in 0·05 mol/l NaCl 0·01 mol/l phosphate buffer, pH 7·3, 1 mmol/l $ZnCl_2$. Cells were then disrupted with an Ultra-Turrax homogenizer and diluted 1 : 1 with 60% (w/v) sucrose and centrifuged at 400 *g* for 30 min over a cushion of 45% sucrose in $H_2O$. The upper layer was harvested and spun at 25,000 *g* for 25 min. The pellet, shown by electron microscopy to contain large plasma membrane vesicles mainly contaminated with endoplasmic reticulum, was suspended in 10 volumes of

* Present address: Laboratory of Immunology, National Institute of Allergy and Infectious Diseases, National Institutes of Health, Bethesda, Maryland 20014, U.S.A.
This work was supported by the Italian National Research Council (C.N.R.).

1% sodium deoxycholate (DOC) in barbitone buffer 0·05 mol/l, pH 8·2 for 20 min. Particulate materials were eliminated by centrifugation at 100,000 *g* for 2 h. The supernatants, designated as solubilized membrane antigens (SMA), were dialysed overnight against a 0·1 DOC solution. Protein concentration was determined by the Lowry *et al.* (1951) method. Radioactive SMA were prepared by acetylation with $^3$H-acetic anhydride (specific activity 500 $\mu$Ci/mmol) by small scale modification of the technique of Agrawal *et al.* (1968). The tumour bearer serum was obtained by pooling sera from animals with tumours 1–1·5 cm diameter and kept at −40°C until required.

### RESULTS

DOC solubilized membrane antigens from both tumours did retain their immunological properties, as shown by their ability to induce and to react with the corresponding specific antibodies (Comoglio and Forni, 1973; Bertini, Forni and Comoglio, 1974). Six groups of 20 mice were inoculated every 48 h for 30 days with sera or SMA, or both, as shown in the Table. With this inoculation schedule a mean circulating dose of 1 ± 0·1 mg/ml SMA was obtained, as determined from the calculation of the half-life of SMA labelled with $^3$H-acetic anhydride. A similar scheme and the same doses were adopted with ADK-lt.

It can be seen from Fig. 1 that neither tumour take nor growth rate was influenced in any way by repeated injection of MOPC-460 tumour bearer serum or the presence of MOPC-460 SMA in the circulating blood. The result was the same even when the bearer serum and MOPC-460 SMA were administered simultaneously. Figure 2 reveals a similar picture for the ADK-lt experiment. Here the tumour take percentage was reduced to a value of 50%, to increase the sensitivity of the system.

Inoculation of treble doses of serum and/or antigen also failed to show differences between the various groups in further experiments using groups of 6 animals and testing both tumours.

### DISCUSSION

The results indicate that the pre-existence and the passive administration of serum from tumour bearing mice or neoplastic cell membrane solubilized antigens, whether singly or in association, neither enhance nor depress the percentage of takes or the growth rate of two transplantable tumours in previously non-immunized mice.

The tested tumours possess tumour specific antigens (Comoglio and Forni, 1973; Bertini, Forni and Comoglio, 1974), some of which can be employed to induce resistance (Lynch *et al.*, 1972; Cavallo and Forni, 1974). Earlier work has also indicated that the "take" of these tumours is influenced by spontaneous or artificially provoked changes in host immune reactivity, suggesting that their growth is hindered by a self-induced mechanism of immunological type (Forni and Comoglio, 1973). The pre-existence

TABLE —*Inoculation Pattern* in Mice Challenged with MOPC*-460

| Group | No. of mice | Inoculum | Challenge with |
|---|---|---|---|
| A | 20 | 0·3 ml normal mouse serum | 2 × 10$^5$ MOPC–460 living cells |
| B | 20 | 0·3 ml MOPC–460 bearer mouse serum | 2 × 10$^5$ MOPC–460 living cells |
| C | 20 | 30 mg normal liver SMA | 2 × 10$^5$ MOPC–460 living cells |
| D | 20 | 30 mg MOPC–460 SMA | 2 × 10$^5$ MOPC–460 living cells |
| E | 20 | 0·3 ml normal mouse serum preincubated with 30 mg MOPC–460 SMA† | 2 × 10$^5$ MOPC–460 living cells |
| F | 20 | 0·3 ml MOPC–460 bearer mouse serum preincubated with 30 mg MOPC–460 SMA† | 2 × 10$^5$ MOPC–460 living cells |

* Inoculations were started 24 h before the challenge with neoplastic cells and were repeated at 48 h intervals for 30 days.

† Pre-incubation at 37°C for 10 min before inoculation.

FIG. 1.—Tumour incidence (A) and growth (B) in mice injected with 2 × $10^5$ MOPC–460 cells. Groups of 20 mice inoculated every 48th h with: (○) 0·3 ml normal mouse serum; (●) 0·3 ml MOPC–460 bearer mouse serum; (□) 30 mg normal liver SMA; (■) 30 mg MOPC–460 SMA; (△) 0·3 ml normal mouse serum preincubated with 30 mg normal liver SMA; (▲) 0·3 ml MOPC–460 bearer mouse serum preincubated with 30 mg MOPC–460 SMA. Vertical lines: ±standard deviation. Hatched area: mean values ± standard deviation for groups inoculated with normal serum and/or normal liver SMA.

FIG. 2.—Tumour incidence (A) and growth (B) in mice injected with 7 × $10^4$ ADK-lt cells. Groups of 20 mice inoculated every 48th h with: (○) 0·3 ml normal mouse serum; (●) 0·3 ml ADK-lt bearer mouse serum; (□) 30 mg normal liver SMA; (■) 30 mg ADK-lt SMA; (△) 0·3 ml normal mouse serum preincubated with 30 mg normal liver SMA; (▲) 0·3 ml ADK-lt bearer mouse serum preincubated with 30 mg ADK-lt SMA. Vertical lines: ± standard deviation. Hatched area: mean values ± standard deviation for groups inoculated with normal serum and/or normal liver SMA.

of serum factors during the first, critical period of the tumour–host relationship has no appreciable effect in situations of this kind, which are comparable with many occurring naturally *in vivo.*

These findings conflict with those obtained in the *in vitro* system, where sensitized lymphocyte activity is inhibited by soluble factors. Failure of these substances to prove effective in our experiment could be due to employment of doses lying outside some narrow and critical concentration range (Baldwin *et al.*, 1972, 1973). Dependence on concentration should, however, be less dominant *in vivo* since the blocking effect is maintained as the relation between antigen and antibodies varies due to increase in the neoplastic mass. Sub-threshold activity would appear to be excluded by the fact that no differences were observed when the challenge dose was altered, or when administration doses were trebled.

Alternatively, the challenge dose could be sufficient to induce the presence of circulating blocking factors and all-or-nothing enhancement of tumour growth. The immunological relation between the host and the developing tumour would thus be unaffected by the administration of further serum factors.

Other explanations may, of course, be forthcoming. Nevertheless, it may fairly be suspected from our findings that serum blocking factors, in spite of their undeniable effect *in vitro* and certain *in vivo* contexts, may often fail to produce significant changes in the naturally evolving development of host resistance to tumour growth.

### REFERENCES

Agrawal, B. B., Goldstein, I. J., Hassing, G. S. & So, L. L. (1968) Protein–Carbohydrate Interaction XVIII. The Preparation and Properties of Acethylated Concanavalin-A, The Haemagglutinin of Jack Bean. *Biochemistry*, **7**, 4211.

Baldwin, R. W., Price, M. R. & Robins, R. A. (1972) Blocking of Lymphocyte-mediated Cytotoxicity for Rat Hepatoma Cells by Tumor-specific Antigen Antibody Complexes. *Nature, New Biol.*, **238**, 185.

Baldwin, R. W., Price, M. A. & Robins, R. A. (1973) Inhibition of Hepatoma-immune Lymph-node Cell Cytotoxicity by Tumor-bearer Serum and Solubilised Hepatoma Antigen. *Int. J. Cancer*, **11**, 527.

Bansal, S. C., Hargreaves, R. & Sjögren, H. O. (1972) Facilitation of Polyoma Tumor Growth in Rats by Blocking Sera and Tumor Eluate. *Int. J. Cancer*, **9**, 97.

Bertini, M., Forni, G. & Comoglio, P. M. (1974) A Tumour-associated Membrane Antigen Transiently Expressed by Normal Cells During Mitosis. *Clin. & exp. Immunol.* In the press.

Cavallo, G. & Forni, G. (1974) Cell Reactivity Towards Syngeneic Neoplastic Cells in Mice Hypersensitized to Dinitrophenol. *Eur. J. Cancer.*, **10**, 103.

Comoglio, P. M. & Forni, G. (1973) Plasma Cells and Tumor Associated Membrane Antigens of Mouse Plasmacytoma MOPC-315 and MOPC-460. *Int. J. Cancer*, **12**, 613.

Currie, G. A. & Basham, C. (1972) Serum Mediated Inhibition of the Immunological Reactions of the Patient to His Own Tumour: A Possible Role for Circulating Antigen. *Br. J. Cancer*, **26**, 427.

Forni, G. & Comoglio, P. M. (1973) Growth of Syngeneic Tumours in Unimmunized Newborn and Adult Hosts. *Br. J. Cancer*, **27**, 120.

Hellström, I. & Hellström, K. E. (1969) Studies of Cellular Immunity and its Serum-mediated Inhibition in Moloney-virus Induced Mouse Sarcomas. *Int. J. Cancer*, **4**, 587.

Hellström, K. E. & Hellström, I. (1974) Lymphocyte-mediated Cytotoxicity and Blocking Serum Activity to Tumor Antigens. *Adv. Immunol.*, **18**, 209.

Lowry, O. H., Rosebrough, N. J., Farr, A. L. & Randall, R. J. (1951) Protein Measurement with Folin Phenol Reagent. *J. biol. Chem.*, **139**, 265.

Lynch, R. G., Graff, R. J., Sirisinha, S., Simms, E. S. & Eisen, H. (1972) Myeloma Proteins as Tumor-specific Transplantation Antigens. *Proc. natn. Acad. Sci. U.S.A.*, **69**, 1540.

Pierce, G. E. (1971) Enhanced Growth of Primary Moloney Virus-induced Sarcomas in Mice. *Int. J. Cancer*, **8**, 22.

Potter, M. (1967) The Plasma Cell Tumours and Myeloma Proteins of Mice. In *Methods in Cancer Research.* Ed. H. Bush, London–New York: Academic Press, Vol. II. p. 105.

Sjögren, S. O., Hellström, I., Bansal, S. C. & Hellström, K. E. (1971) Suggestive Evidence that the " Blocking Antibodies " of Tumor Bearing Individuals may be Antigen–Antibody Complexes. *Proc. natn. Acad. Sci. U.S.A.*, **68**, 1372.

Thomson, D. M. P., Eccles, S. & Alexander, P. (1973) Antibodies and Soluble Tumour Specific Antigens in Blood and Lymph of Rats with Chemically Induced Sarcomata. *Br. J. Cancer*, **28**, 6.

Vaage, J. (1973) Humoral and Cellular Immune Foctors in the Systemic Control of Artificially Induced Metastases in C3HF Mice. *Cancer Res.*, **33**, 1957.

Voisin, G. A. (1971) Immunological Facilitation, a Broadening of the Concept of the Enhancement Phenomenon. *Prog. Allergy*, **15**, 328.